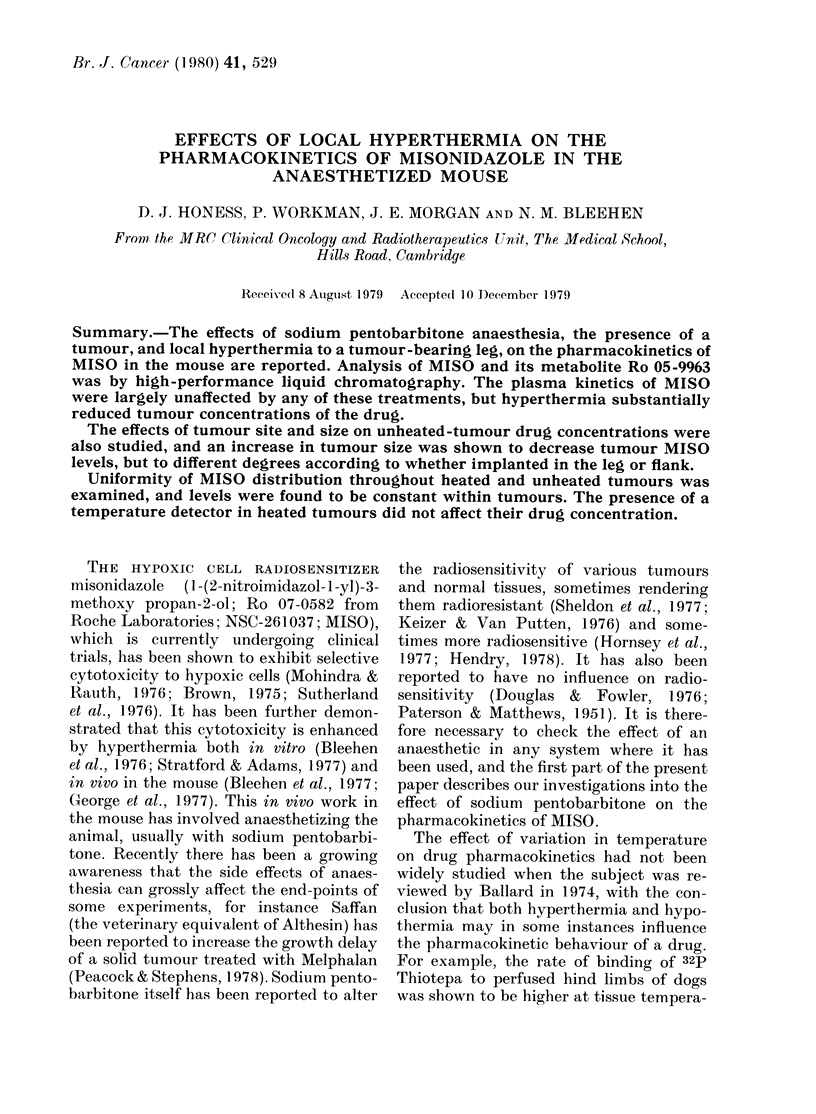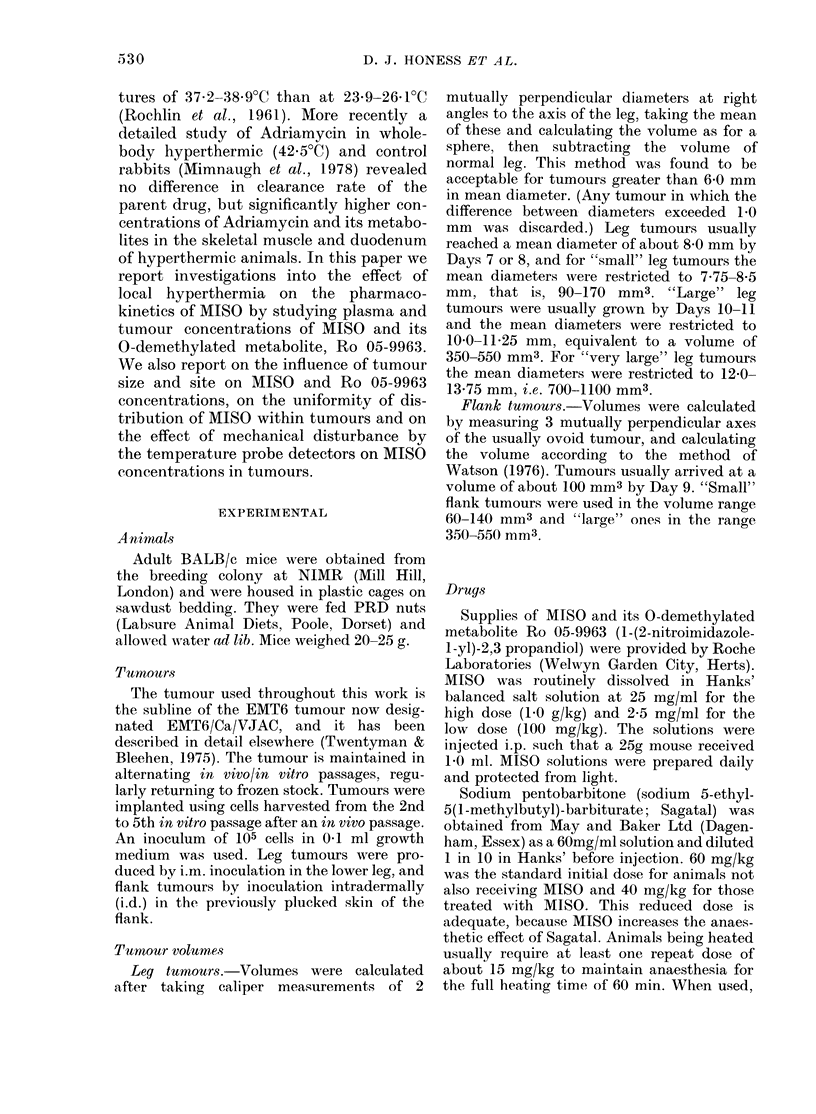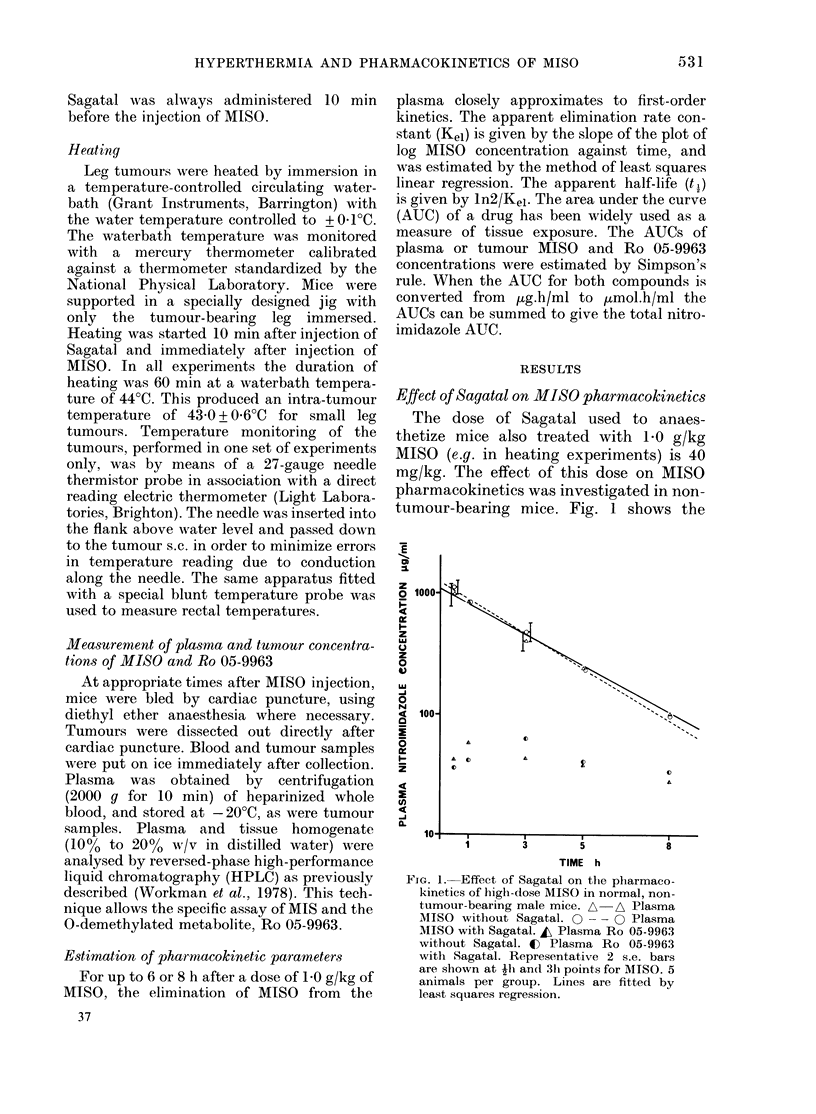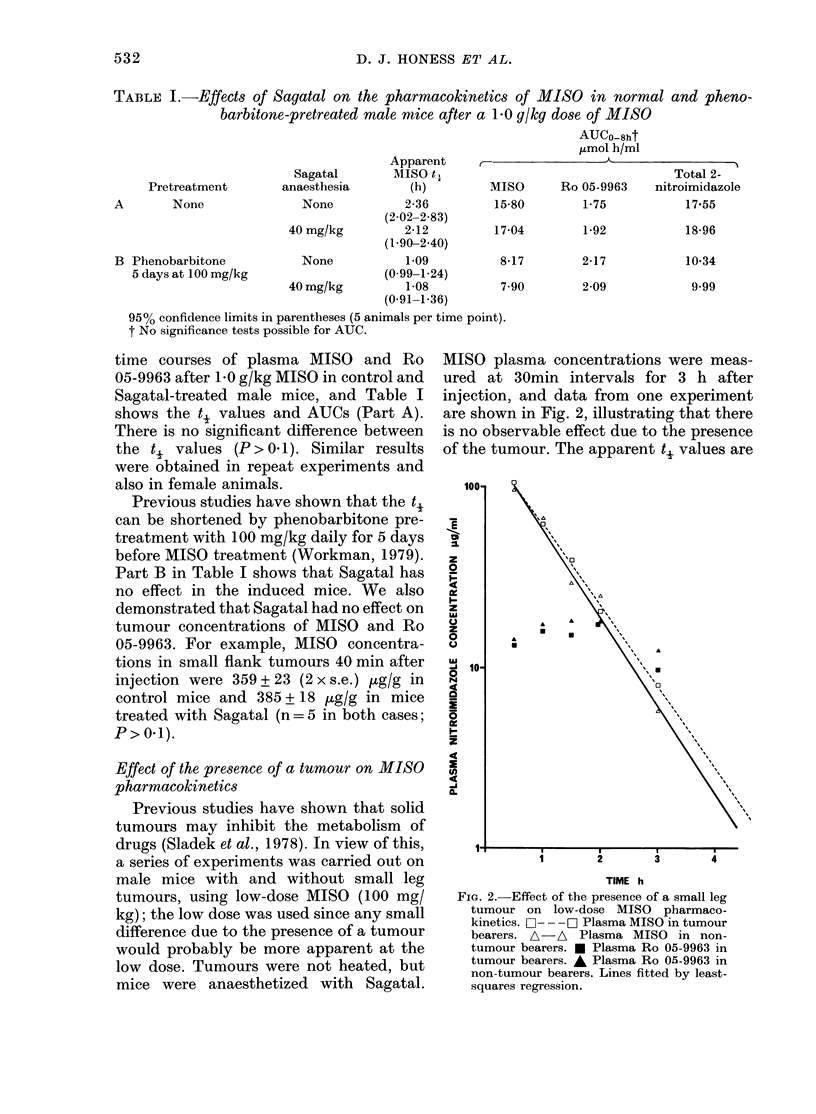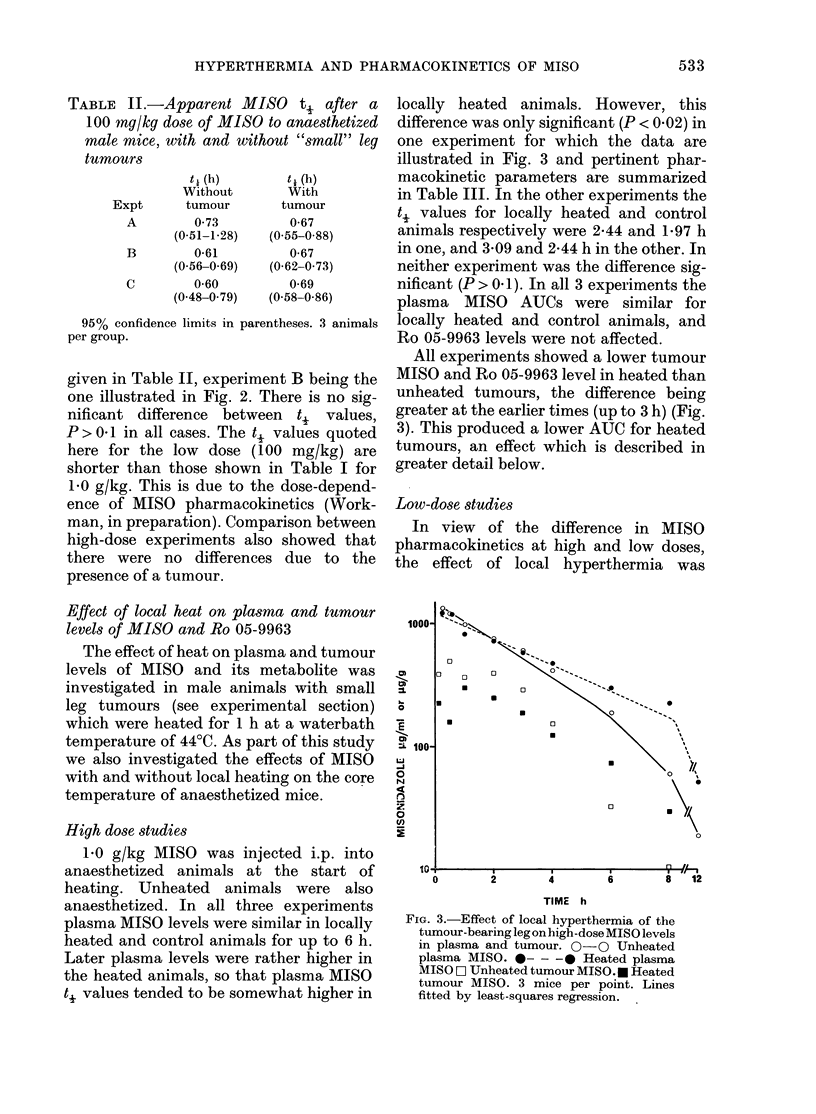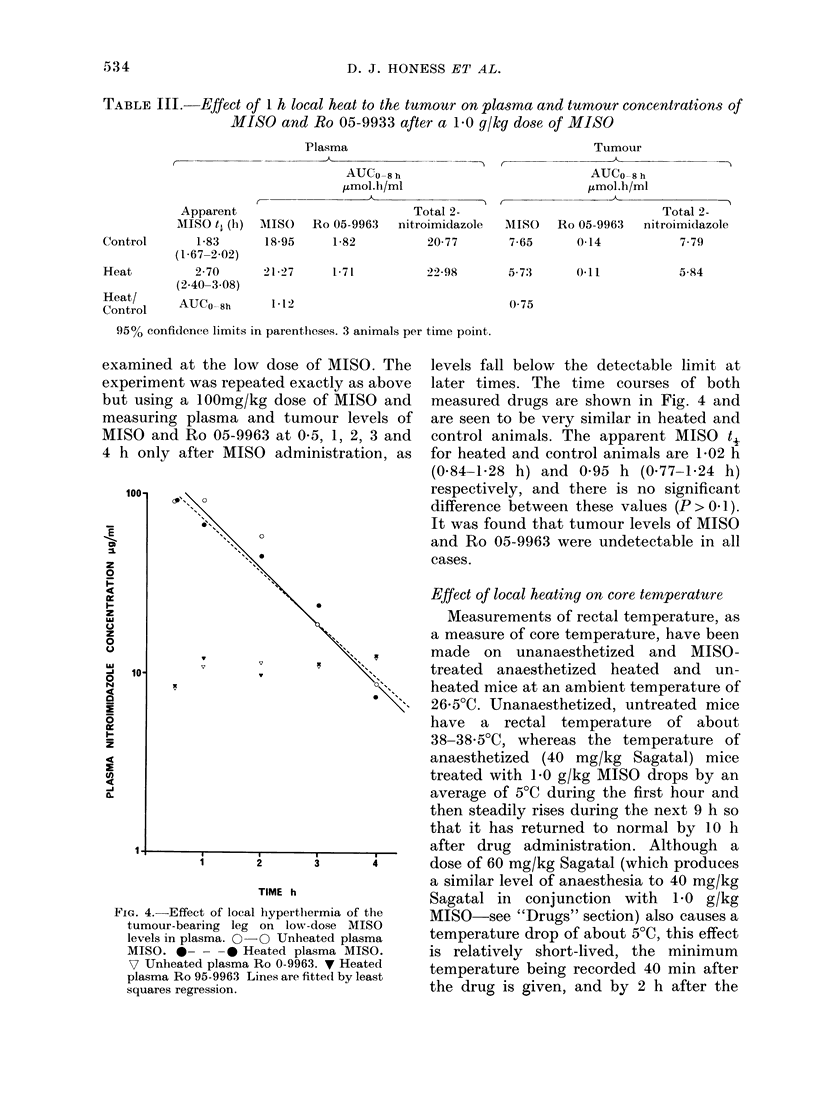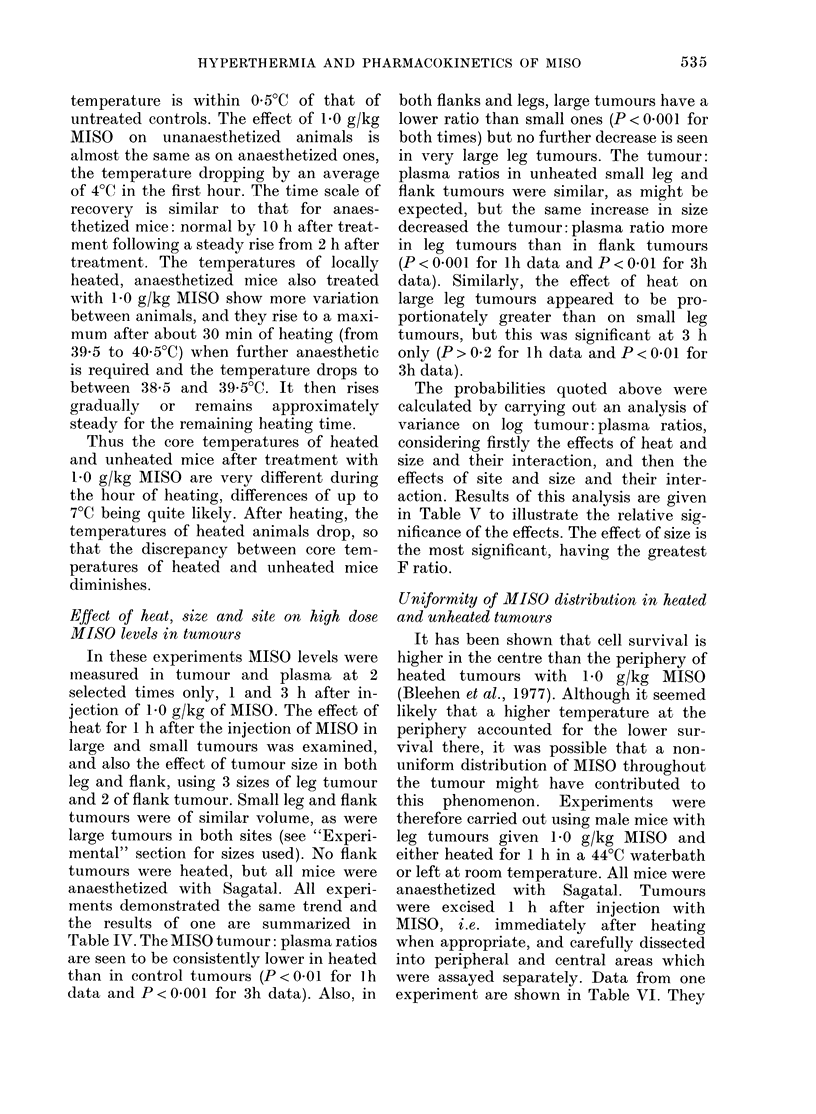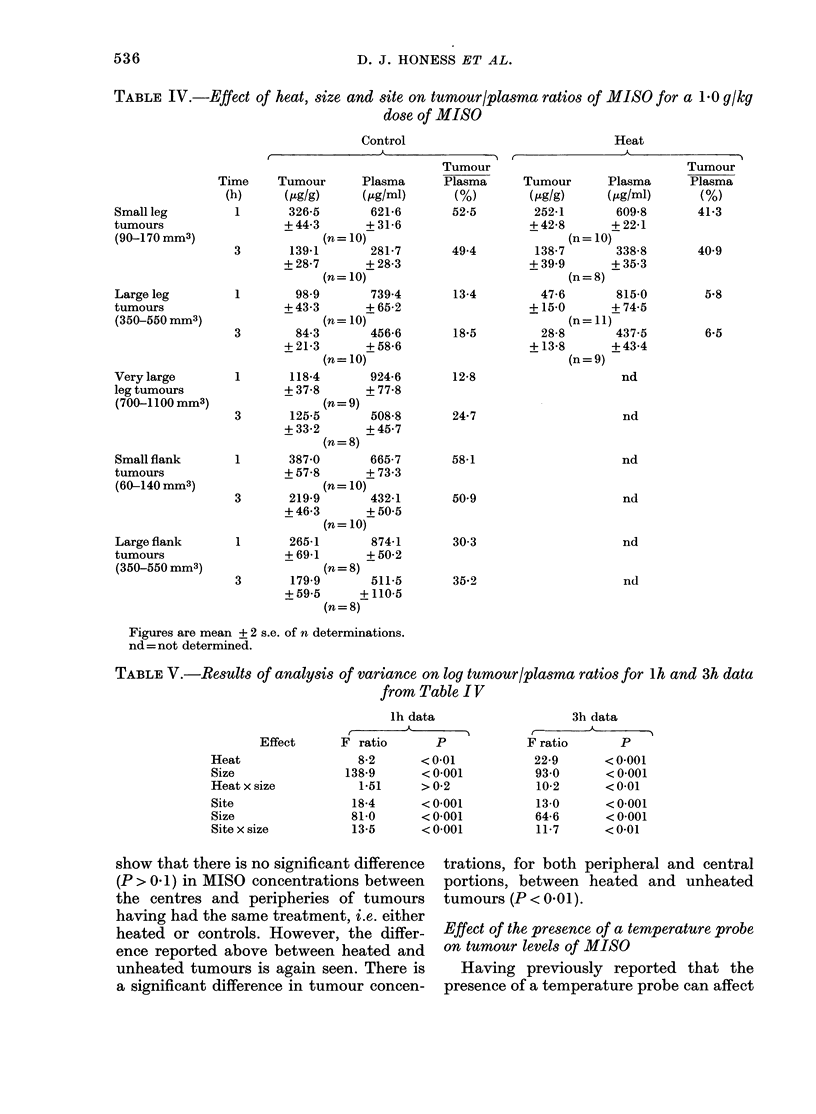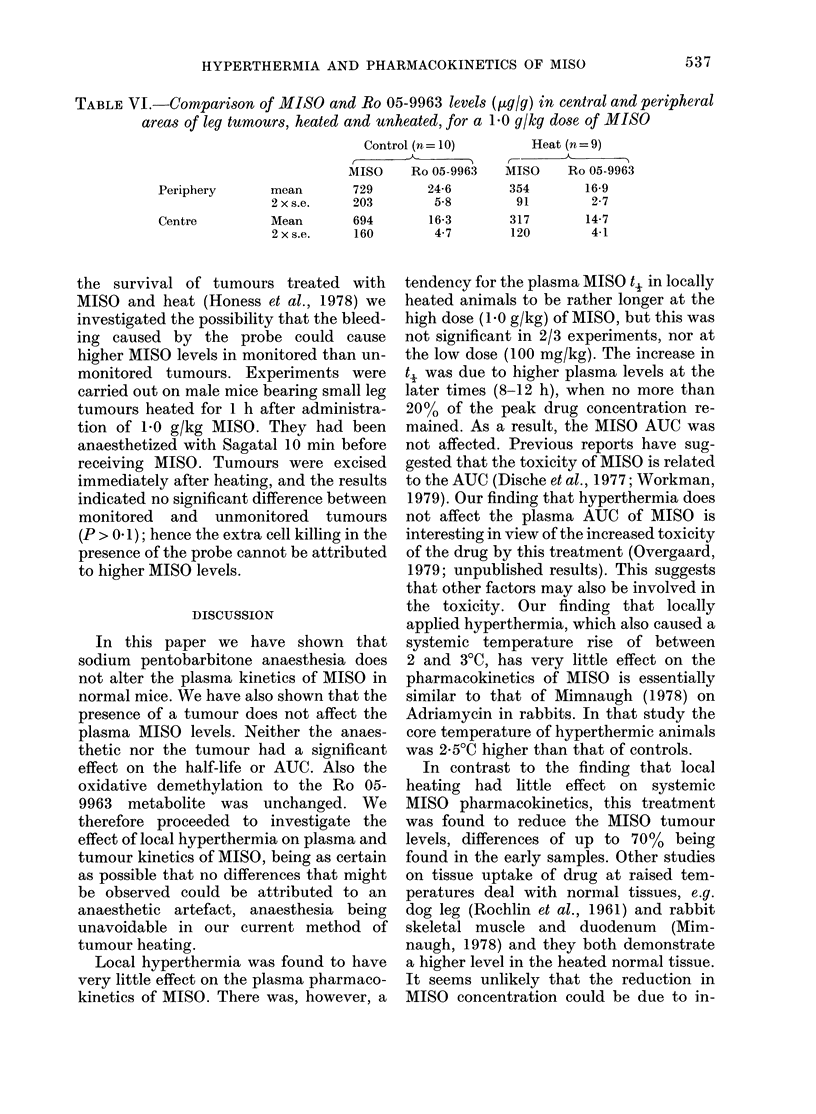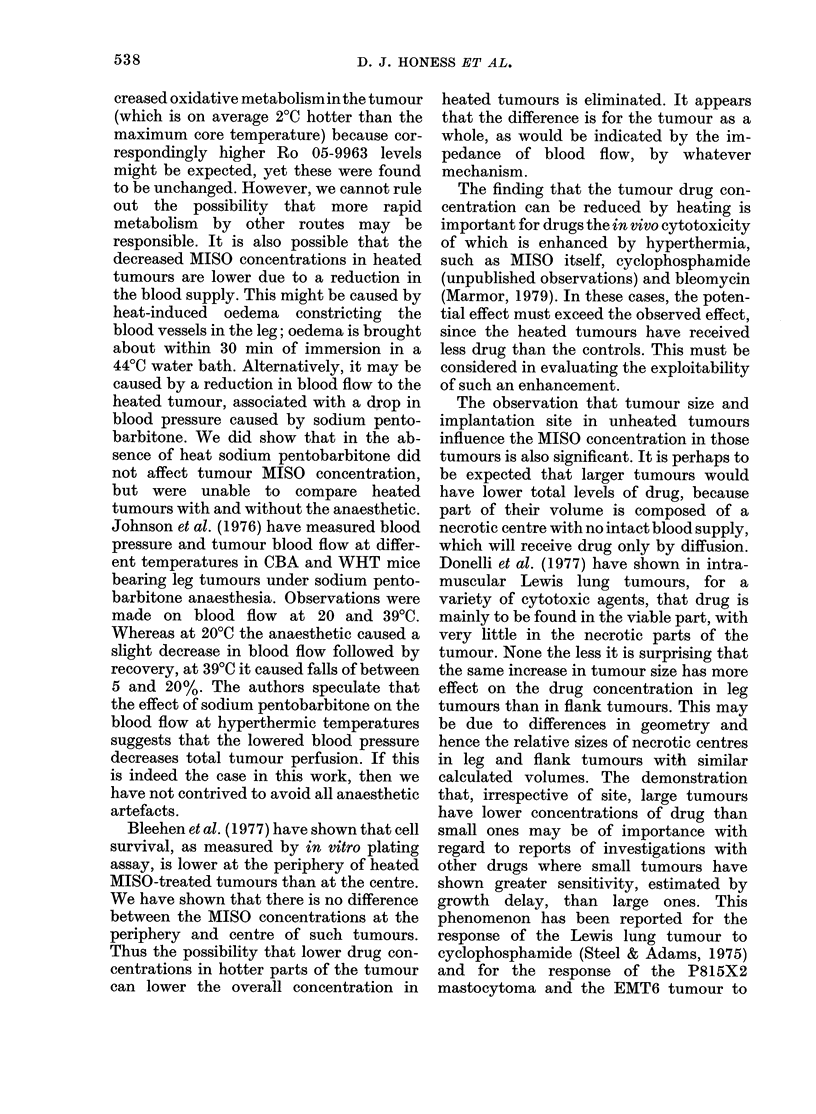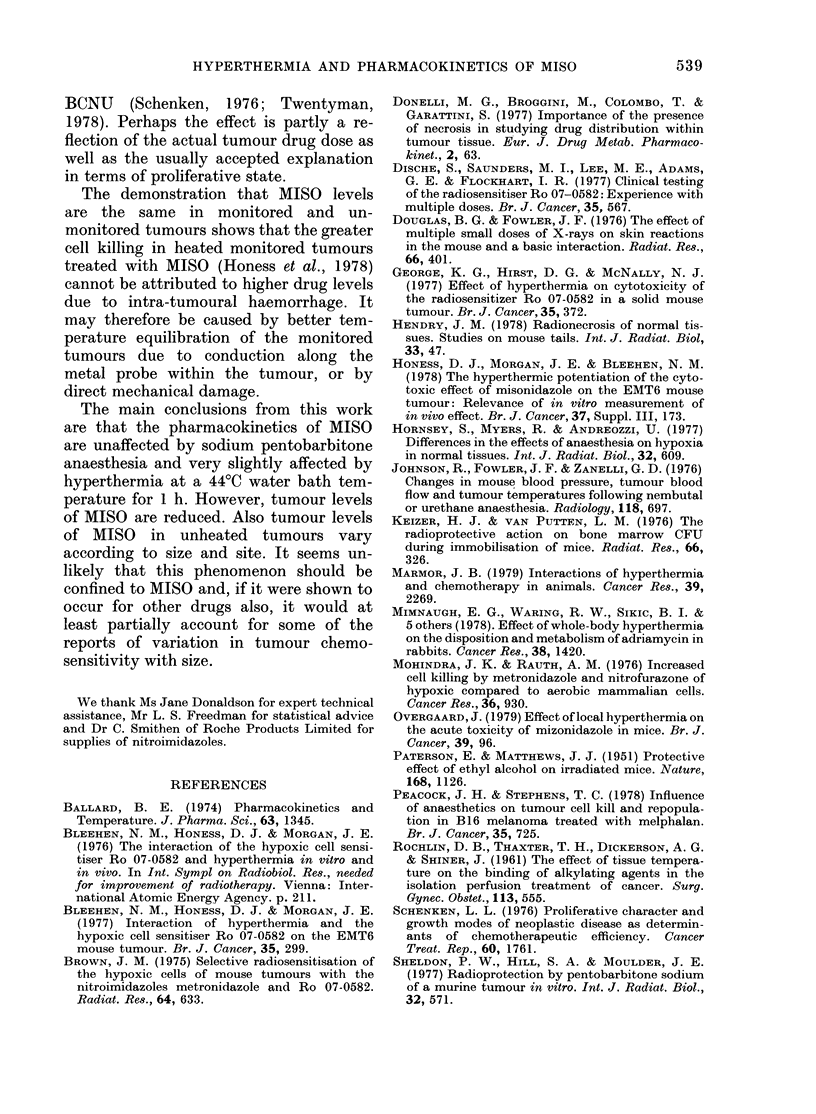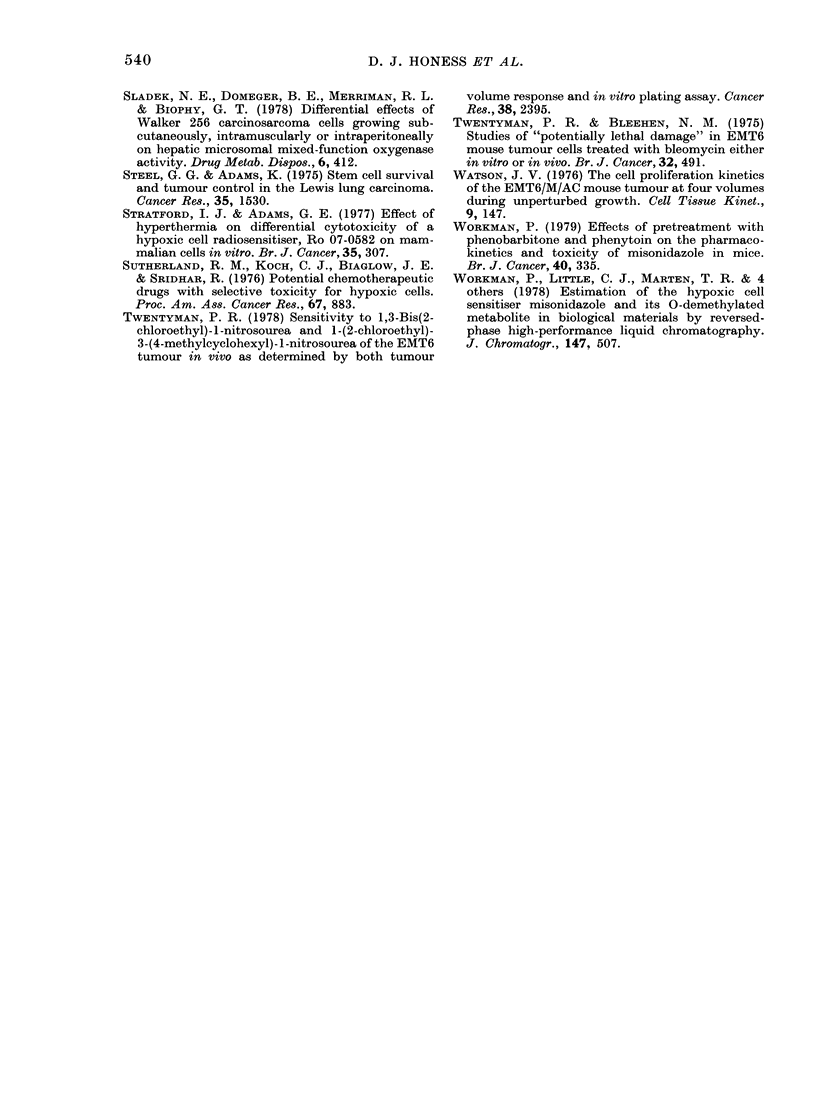# Effects of local hyperthermia on the pharmacokinetics of misonidazole in the anaesthetized mouse.

**DOI:** 10.1038/bjc.1980.95

**Published:** 1980-04

**Authors:** D. J. Honess, P. Workman, J. E. Morgan, N. M. Bleehen

## Abstract

The effects of sodium pentobarbitone anaesthesia, the presence of a tumour, and local hyperthermia to a tumour-bearing leg, on the pharmacokinetics of MISO in the mouse are reported. Analysis of MISO and its metabolite Ro 05-9963 was by high-performance liquid chromatography. The plasma kinetics of MISO were largely unaffected by any of these treatments, but hyperthermia substantially reduced tumour concentrations of the drug. The effects of tumour site and size on unheated-tumour drug concentrations were also studied, and an increase in tumour size was shown to decrease tumour MISO levels, but to different degrees according to whether implanted in the leg or flank. Uniformity of MISO distribution throughout heated and unheated tumours was examined, and levels were found to be constant within tumours. The presence of a temperature detector in heated tumours did not affect their drug concentration.


*Br. J. Cancer* (1980) **41**, 529

### EFFECTS OF LOCAL HYPERTHERMIA ON THE PHARMACOKINETICS OF MISONIDAZOLE IN THE ANAESTHETIZED MOUSE

D. J. HONESS, P. WORKMAN, J. E. MORGAN AND N. M. BLEEHEN

*From the MRC Clinical Oncology and Radiotherapeutics Unit, The Medical School, Hills Road, Cambridge*

Received 8 August 1979 Accepted 10 December 1979

**Summary.—The effects of sodium pentobarbitone anaesthesia, the presence of a tumour, and local hyperthermia to a tumour-bearing leg, on the pharmacokinetics of MISO in the mouse are reported. Analysis of MISO and its metabolite Ro 05-9963 was by high-performance liquid chromatography. The plasma kinetics of MISO were largely unaffected by any of these treatments, but hyperthermia substantially reduced tumour concentrations of the drug.**

**The effects of tumour site and size on unheated-tumour drug concentrations were also studied, and an increase in tumour size was shown to decrease tumour MISO levels, but to different degrees according to whether implanted in the leg or flank.**

**Uniformity of MISO distribution throughout heated and unheated tumours was examined, and levels were found to be constant within tumours. The presence of a temperature detector in heated tumours did not affect their drug concentration.**

THE HYPOXIC CELL RADIOSENSITIZER misonidazole (1-(2-nitroimidazol-1-yl)-3-methoxy propan-2-ol; Ro 07-0582 from Roche Laboratories; NSC-261037; MISO), which is currently undergoing clinical trials, has been shown to exhibit selective cytotoxicity to hypoxic cells (Mohindra & Rauth, 1976; Brown, 1975; Sutherland *et al.*, 1976). It has been further demonstrated that this cytotoxicity is enhanced by hyperthermia both *in vitro* (Bleehen *et al.*, 1976; Stratford & Adams, 1977) and *in vivo* in the mouse (Bleehen *et al.*, 1977; George *et al.*, 1977). This *in vivo* work in the mouse has involved anaesthetizing the animal, usually with sodium pentobarbitone. Recently there has been a growing awareness that the side effects of anaesthesia can grossly affect the end-points of some experiments, for instance Saffan (the veterinary equivalent of Althesin) has been reported to increase the growth delay of a solid tumour treated with Melphalan (Peacock & Stephens, 1978). Sodium pentobarbitone itself has been reported to alter the radiosensitivity of various tumours and normal tissues, sometimes rendering them radioresistant (Sheldon *et al.*, 1977; Keizer & Van Putten, 1976) and sometimes more radiosensitive (Hornsey *et al.*, 1977; Hendry, 1978). It has also been reported to have no influence on radiosensitivity (Douglas & Fowler, 1976; Paterson & Matthews, 1951). It is therefore necessary to check the effect of an anaesthetic in any system where it has been used, and the first part of the present paper describes our investigations into the effect of sodium pentobarbitone on the pharmacokinetics of MISO.

The effect of variation in temperature on drug pharmacokinetics had not been widely studied when the subject was reviewed by Ballard in 1974, with the conclusion that both hyperthermia and hypothermia may in some instances influence the pharmacokinetic behaviour of a drug. For example, the rate of binding of $^{32}$P Thiotepa to perfused hind limbs of dogs was shown to be higher at tissue temperatures of 37·2–38·9°C than at 23·9–26·1°C (Rochlin *et al.*, 1961). More recently a detailed study of Adriamycin in whole-body hyperthermic (42·5°C) and control rabbits (Mimnaugh *et al.*, 1978) revealed no difference in clearance rate of the parent drug, but significantly higher concentrations of Adriamycin and its metabolites in the skeletal muscle and duodenum of hyperthermic animals. In this paper we report investigations into the effect of local hyperthermia on the pharmacokinetics of MISO by studying plasma and tumour concentrations of MISO and its O-demethylated metabolite, Ro 05-9963. We also report on the influence of tumour size and site on MISO and Ro 05-9963 concentrations, on the uniformity of distribution of MISO within tumours and on the effect of mechanical disturbance by the temperature probe detectors on MISO concentrations in tumours.

### EXPERIMENTAL

### *Animals*

Adult BALB/c mice were obtained from the breeding colony at NIMR (Mill Hill, London) and were housed in plastic cages on sawdust bedding. They were fed PRD nuts (Labsure Animal Diets, Poole, Dorset) and allowed water *ad lib.* Mice weighed 20–25 g.

### *Tumours*

The tumour used throughout this work is the subline of the EMT6 tumour now designated EMT6/Ca/VJAC, and it has been described in detail elsewhere (Twentyman & Bleehen, 1975). The tumour is maintained in alternating *in vivo*/*in vitro* passages, regularly returning to frozen stock. Tumours were implanted using cells harvested from the 2nd to 5th *in vitro* passage after an *in vivo* passage. An inoculum of $10^5$ cells in 0·1 ml growth medium was used. Leg tumours were produced by i.m. inoculation in the lower leg, and flank tumours by inoculation intradermally (i.d.) in the previously plucked skin of the flank.

### *Tumour volumes*

*Leg tumours.*—Volumes were calculated after taking caliper measurements of 2 mutually perpendicular diameters at right angles to the axis of the leg, taking the mean of these and calculating the volume as for a sphere, then subtracting the volume of normal leg. This method was found to be acceptable for tumours greater than 6·0 mm in mean diameter. (Any tumour in which the difference between diameters exceeded 1·0 mm was discarded.) Leg tumours usually reached a mean diameter of about 8·0 mm by Days 7 or 8, and for "small" leg tumours the mean diameters were restricted to 7·75–8·5 mm, that is, 90–170 mm$^3$. "Large" leg tumours were usually grown by Days 10–11 and the mean diameters were restricted to 10·0–11·25 mm, equivalent to a volume of 350–550 mm$^3$. For "very large" leg tumours the mean diameters were restricted to 12·0–13·75 mm, *i.e.* 700–1100 mm$^3$.

*Flank tumours.*—Volumes were calculated by measuring 3 mutually perpendicular axes of the usually ovoid tumour, and calculating the volume according to the method of Watson (1976). Tumours usually arrived at a volume of about 100 mm$^3$ by Day 9. "Small" flank tumours were used in the volume range 60–140 mm$^3$ and "large" ones in the range 350–550 mm$^3$.

### *Drugs*

Supplies of MISO and its O-demethylated metabolite Ro 05-9963 (1-(2-nitroimidazole-1-yl)-2,3 propandiol) were provided by Roche Laboratories (Welwyn Garden City, Herts). MISO was routinely dissolved in Hanks' balanced salt solution at 25 mg/ml for the high dose (1·0 g/kg) and 2·5 mg/ml for the low dose (100 mg/kg). The solutions were injected i.p. such that a 25g mouse received 1·0 ml. MISO solutions were prepared daily and protected from light.

Sodium pentobarbitone (sodium 5-ethyl-5(1-methylbutyl)-barbiturate; Sagatal) was obtained from May and Baker Ltd (Dagenham, Essex) as a 60mg/ml solution and diluted 1 in 10 in Hanks' before injection. 60 mg/kg was the standard initial dose for animals not also receiving MISO and 40 mg/kg for those treated with MISO. This reduced dose is adequate, because MISO increases the anaesthetic effect of Sagatal. Animals being heated usually require at least one repeat dose of about 15 mg/kg to maintain anaesthesia for the full heating time of 60 min. When used,

Sagatal was always administered 10 min before the injection of MISO.

*Heating*

Leg tumours were heated by immersion in a temperature-controlled circulating water-bath (Grant Instruments, Barrington) with the water temperature controlled to $\pm 0{\cdot}1°C$. The waterbath temperature was monitored with a mercury thermometer calibrated against a thermometer standardized by the National Physical Laboratory. Mice were supported in a specially designed jig with only the tumour-bearing leg immersed. Heating was started 10 min after injection of Sagatal and immediately after injection of MISO. In all experiments the duration of heating was 60 min at a waterbath temperature of 44°C. This produced an intra-tumour temperature of $43{\cdot}0 \pm 0{\cdot}6°C$ for small leg tumours. Temperature monitoring of the tumours, performed in one set of experiments only, was by means of a 27-gauge needle thermistor probe in association with a direct reading electric thermometer (Light Laboratories, Brighton). The needle was inserted into the flank above water level and passed down to the tumour s.c. in order to minimize errors in temperature reading due to conduction along the needle. The same apparatus fitted with a special blunt temperature probe was used to measure rectal temperatures.

*Measurement of plasma and tumour concentrations of MISO and Ro* 05-9963

At appropriate times after MISO injection, mice were bled by cardiac puncture, using diethyl ether anaesthesia where necessary. Tumours were dissected out directly after cardiac puncture. Blood and tumour samples were put on ice immediately after collection. Plasma was obtained by centrifugation (2000 *g* for 10 min) of heparinized whole blood, and stored at −20°C, as were tumour samples. Plasma and tissue homogenate (10% to 20% w/v in distilled water) were analysed by reversed-phase high-performance liquid chromatography (HPLC) as previously described (Workman *et al.*, 1978). This technique allows the specific assay of MIS and the O-demethylated metabolite, Ro 05-9963.

*Estimation of pharmacokinetic parameters*

For up to 6 or 8 h after a dose of 1·0 g/kg of MISO, the elimination of MISO from the plasma closely approximates to first-order kinetics. The apparent elimination rate constant ($K_{el}$) is given by the slope of the plot of log MISO concentration against time, and was estimated by the method of least squares linear regression. The apparent half-life ($t_{\frac{1}{2}}$) is given by $\ln 2/K_{el}$. The area under the curve (AUC) of a drug has been widely used as a measure of tissue exposure. The AUCs of plasma or tumour MISO and Ro 05-9963 concentrations were estimated by Simpson's rule. When the AUC for both compounds is converted from $\mu$g.h/ml to $\mu$mol.h/ml the AUCs can be summed to give the total nitroimidazole AUC.

### RESULTS

*Effect of Sagatal on MISO pharmacokinetics*

The dose of Sagatal used to anaesthetize mice also treated with 1·0 g/kg MISO (*e.g.* in heating experiments) is 40 mg/kg. The effect of this dose on MISO pharmacokinetics was investigated in non-tumour-bearing mice. Fig. 1 shows the

FIG. 1.—Effect of Sagatal on the pharmacokinetics of high-dose MISO in normal, non-tumour-bearing male mice. △—△ Plasma MISO without Sagatal. ○ – – ○ Plasma MISO with Sagatal. ▲ Plasma Ro 05-9963 without Sagatal. ◐ Plasma Ro 05-9963 with Sagatal. Representative 2 s.e. bars are shown at ½h and 3h points for MISO. 5 animals per group. Lines are fitted by least squares regression.

TABLE I.—*Effects of Sagatal on the pharmacokinetics of MISO in normal and phenobarbitone-pretreated male mice after a* 1·0 *g/kg dose of MISO*

| | Pretreatment | Sagatal anaesthesia | Apparent MISO $t_{\frac{1}{2}}$ (h) | AUC$_{0-8h}$† μmol h/ml MISO | Ro 05-9963 | Total 2-nitroimidazole |
|---|---|---|---|---|---|---|
| A | None | None | 2·36 (2·02–2·83) | 15·80 | 1·75 | 17·55 |
| | | 40 mg/kg | 2·12 (1·90–2·40) | 17·04 | 1·92 | 18·96 |
| B | Phenobarbitone 5 days at 100 mg/kg | None | 1·09 (0·99–1·24) | 8·17 | 2·17 | 10·34 |
| | | 40 mg/kg | 1·08 (0·91–1·36) | 7·90 | 2·09 | 9·99 |

95% confidence limits in parentheses (5 animals per time point).
† No significance tests possible for AUC.

time courses of plasma MISO and Ro 05-9963 after 1·0 g/kg MISO in control and Sagatal-treated male mice, and Table I shows the $t_{\frac{1}{2}}$ values and AUCs (Part A). There is no significant difference between the $t_{\frac{1}{2}}$ values ($P > 0{\cdot}1$). Similar results were obtained in repeat experiments and also in female animals.

Previous studies have shown that the $t_{\frac{1}{2}}$ can be shortened by phenobarbitone pretreatment with 100 mg/kg daily for 5 days before MISO treatment (Workman, 1979). Part B in Table I shows that Sagatal has no effect in the induced mice. We also demonstrated that Sagatal had no effect on tumour concentrations of MISO and Ro 05-9963. For example, MISO concentrations in small flank tumours 40 min after injection were 359 ± 23 (2 × s.e.) μg/g in control mice and 385 ± 18 μg/g in mice treated with Sagatal (n = 5 in both cases; $P > 0{\cdot}1$).

*Effect of the presence of a tumour on MISO pharmacokinetics*

Previous studies have shown that solid tumours may inhibit the metabolism of drugs (Sladek *et al.*, 1978). In view of this, a series of experiments was carried out on male mice with and without small leg tumours, using low-dose MISO (100 mg/kg); the low dose was used since any small difference due to the presence of a tumour would probably be more apparent at the low dose. Tumours were not heated, but mice were anaesthetized with Sagatal. MISO plasma concentrations were measured at 30min intervals for 3 h after injection, and data from one experiment are shown in Fig. 2, illustrating that there is no observable effect due to the presence of the tumour. The apparent $t_{\frac{1}{2}}$ values are

FIG. 2.—Effect of the presence of a small leg tumour on low-dose MISO pharmacokinetics. □- - -□ Plasma MISO in tumour bearers. △—△ Plasma MISO in non-tumour bearers. ■ Plasma Ro 05-9963 in tumour bearers. ▲ Plasma Ro 05-9963 in non-tumour bearers. Lines fitted by least-squares regression.

TABLE II.—*Apparent MISO* $t_{\frac{1}{2}}$ *after a* 100 *mg/kg dose of MISO to anaesthetized male mice, with and without "small" leg tumours*

| Expt | $t_{\frac{1}{2}}$ (h) Without tumour | $t_{\frac{1}{2}}$ (h) With tumour |
|---|---|---|
| A | 0·73 (0·51–1·28) | 0·67 (0·55–0·88) |
| B | 0·61 (0·56–0·69) | 0·67 (0·62–0·73) |
| C | 0·60 (0·48–0·79) | 0·69 (0·58–0·86) |

95% confidence limits in parentheses. 3 animals per group.

given in Table II, experiment B being the one illustrated in Fig. 2. There is no significant difference between $t_{\frac{1}{2}}$ values, $P > 0·1$ in all cases. The $t_{\frac{1}{2}}$ values quoted here for the low dose (100 mg/kg) are shorter than those shown in Table I for 1·0 g/kg. This is due to the dose-dependence of MISO pharmacokinetics (Workman, in preparation). Comparison between high-dose experiments also showed that there were no differences due to the presence of a tumour.

*Effect of local heat on plasma and tumour levels of MISO and Ro* 05-9963

The effect of heat on plasma and tumour levels of MISO and its metabolite was investigated in male animals with small leg tumours (see experimental section) which were heated for 1 h at a waterbath temperature of 44°C. As part of this study we also investigated the effects of MISO with and without local heating on the core temperature of anaesthetized mice.

*High dose studies*

1·0 g/kg MISO was injected i.p. into anaesthetized animals at the start of heating. Unheated animals were also anaesthetized. In all three experiments plasma MISO levels were similar in locally heated and control animals for up to 6 h. Later plasma levels were rather higher in the heated animals, so that plasma MISO $t_{\frac{1}{2}}$ values tended to be somewhat higher in locally heated animals. However, this difference was only significant ($P < 0·02$) in one experiment for which the data are illustrated in Fig. 3 and pertinent pharmacokinetic parameters are summarized in Table III. In the other experiments the $t_{\frac{1}{2}}$ values for locally heated and control animals respectively were 2·44 and 1·97 h in one, and 3·09 and 2·44 h in the other. In neither experiment was the difference significant ($P > 0·1$). In all 3 experiments the plasma MISO AUCs were similar for locally heated and control animals, and Ro 05-9963 levels were not affected.

All experiments showed a lower tumour MISO and Ro 05-9963 level in heated than unheated tumours, the difference being greater at the earlier times (up to 3 h) (Fig. 3). This produced a lower AUC for heated tumours, an effect which is described in greater detail below.

*Low-dose studies*

In view of the difference in MISO pharmacokinetics at high and low doses, the effect of local hyperthermia was

FIG. 3.—Effect of local hyperthermia of the tumour-bearing leg on high-dose MISO levels in plasma and tumour. ○—○ Unheated plasma MISO. ●- - -● Heated plasma MISO □ Unheated tumour MISO. ■ Heated tumour MISO. 3 mice per point. Lines fitted by least-squares regression.

TABLE III.—*Effect of* 1 *h local heat to the tumour on plasma and tumour concentrations of MISO and Ro* 05-9933 *after a* 1·0 *g/kg dose of MISO*

| | | Plasma | | | Tumour | | |
|---|---|---|---|---|---|---|---|
| | | $AUC_{0-8\,h}$ µmol.h/ml | | | $AUC_{0-8\,h}$ µmol.h/ml | | |
| | Apparent MISO $t_{\frac{1}{2}}$ (h) | MISO | Ro 05-9963 | Total 2-nitroimidazole | MISO | Ro 05-9963 | Total 2-nitroimidazole |
| Control | 1·83 (1·67–2·02) | 18·95 | 1·82 | 20·77 | 7·65 | 0·14 | 7·79 |
| Heat | 2·70 (2·40–3·08) | 21·27 | 1·71 | 22·98 | 5·73 | 0·11 | 5·84 |
| Heat/Control | $AUC_{0-8h}$ | 1·12 | | | 0·75 | | |

95% confidence limits in parentheses. 3 animals per time point.

examined at the low dose of MISO. The experiment was repeated exactly as above but using a 100mg/kg dose of MISO and measuring plasma and tumour levels of MISO and Ro 05-9963 at 0·5, 1, 2, 3 and 4 h only after MISO administration, as

FIG. 4.—Effect of local hyperthermia of the tumour-bearing leg on low-dose MISO levels in plasma. ○—○ Unheated plasma MISO. ●– – –● Heated plasma MISO. ▽ Unheated plasma Ro 0-9963. ▼ Heated plasma Ro 95-9963 Lines are fitted by least squares regression.

levels fall below the detectable limit at later times. The time courses of both measured drugs are shown in Fig. 4 and are seen to be very similar in heated and control animals. The apparent MISO $t_{\frac{1}{2}}$ for heated and control animals are 1·02 h (0·84–1·28 h) and 0·95 h (0·77–1·24 h) respectively, and there is no significant difference between these values ($P > 0{\cdot}1$). It was found that tumour levels of MISO and Ro 05-9963 were undetectable in all cases.

*Effect of local heating on core temperature*

Measurements of rectal temperature, as a measure of core temperature, have been made on unanaesthetized and MISO-treated anaesthetized heated and unheated mice at an ambient temperature of 26·5°C. Unanaesthetized, untreated mice have a rectal temperature of about 38–38·5°C, whereas the temperature of anaesthetized (40 mg/kg Sagatal) mice treated with 1·0 g/kg MISO drops by an average of 5°C during the first hour and then steadily rises during the next 9 h so that it has returned to normal by 10 h after drug administration. Although a dose of 60 mg/kg Sagatal (which produces a similar level of anaesthesia to 40 mg/kg Sagatal in conjunction with 1·0 g/kg MISO—see "Drugs" section) also causes a temperature drop of about 5°C, this effect is relatively short-lived, the minimum temperature being recorded 40 min after the drug is given, and by 2 h after the temperature is within 0·5°C of that of untreated controls. The effect of 1·0 g/kg MISO on unanaesthetized animals is almost the same as on anaesthetized ones, the temperature dropping by an average of 4°C in the first hour. The time scale of recovery is similar to that for anaesthetized mice: normal by 10 h after treatment following a steady rise from 2 h after treatment. The temperatures of locally heated, anaesthetized mice also treated with 1·0 g/kg MISO show more variation between animals, and they rise to a maximum after about 30 min of heating (from 39·5 to 40·5°C) when further anaesthetic is required and the temperature drops to between 38·5 and 39·5°C. It then rises gradually or remains approximately steady for the remaining heating time.

Thus the core temperatures of heated and unheated mice after treatment with 1·0 g/kg MISO are very different during the hour of heating, differences of up to 7°C being quite likely. After heating, the temperatures of heated animals drop, so that the discrepancy between core temperatures of heated and unheated mice diminishes.

*Effect of heat, size and site on high dose MISO levels in tumours*

In these experiments MISO levels were measured in tumour and plasma at 2 selected times only, 1 and 3 h after injection of 1·0 g/kg of MISO. The effect of heat for 1 h after the injection of MISO in large and small tumours was examined, and also the effect of tumour size in both leg and flank, using 3 sizes of leg tumour and 2 of flank tumour. Small leg and flank tumours were of similar volume, as were large tumours in both sites (see "Experimental" section for sizes used). No flank tumours were heated, but all mice were anaesthetized with Sagatal. All experiments demonstrated the same trend and the results of one are summarized in Table IV. The MISO tumour: plasma ratios are seen to be consistently lower in heated than in control tumours ($P < 0{\cdot}01$ for 1h data and $P < 0{\cdot}001$ for 3h data). Also, in both flanks and legs, large tumours have a lower ratio than small ones ($P < 0{\cdot}001$ for both times) but no further decrease is seen in very large leg tumours. The tumour: plasma ratios in unheated small leg and flank tumours were similar, as might be expected, but the same increase in size decreased the tumour: plasma ratio more in leg tumours than in flank tumours ($P < 0{\cdot}001$ for 1h data and $P < 0{\cdot}01$ for 3h data). Similarly, the effect of heat on large leg tumours appeared to be proportionately greater than on small leg tumours, but this was significant at 3 h only ($P > 0{\cdot}2$ for 1h data and $P < 0{\cdot}01$ for 3h data).

The probabilities quoted above were calculated by carrying out an analysis of variance on log tumour: plasma ratios, considering firstly the effects of heat and size and their interaction, and then the effects of site and size and their interaction. Results of this analysis are given in Table V to illustrate the relative significance of the effects. The effect of size is the most significant, having the greatest F ratio.

*Uniformity of MISO distribution in heated and unheated tumours*

It has been shown that cell survival is higher in the centre than the periphery of heated tumours with 1·0 g/kg MISO (Bleehen *et al.*, 1977). Although it seemed likely that a higher temperature at the periphery accounted for the lower survival there, it was possible that a non-uniform distribution of MISO throughout the tumour might have contributed to this phenomenon. Experiments were therefore carried out using male mice with leg tumours given 1·0 g/kg MISO and either heated for 1 h in a 44°C waterbath or left at room temperature. All mice were anaesthetized with Sagatal. Tumours were excised 1 h after injection with MISO, *i.e.* immediately after heating when appropriate, and carefully dissected into peripheral and central areas which were assayed separately. Data from one experiment are shown in Table VI. They

TABLE IV.—*Effect of heat, size and site on tumour/plasma ratios of MISO for a* 1·0 *g/kg dose of MISO*

| | | Control | | | Heat | | |
|---|---|---|---|---|---|---|---|
| | Time (h) | Tumour ($\mu$g/g) | Plasma ($\mu$g/ml) | Tumour/Plasma (%) | Tumour ($\mu$g/g) | Plasma ($\mu$g/ml) | Tumour/Plasma (%) |
| Small leg tumours (90–170 mm³) | 1 | 326·5 | 621·6 | 52·5 | 252·1 | 609·8 | 41·3 |
| | | ±44·3 | ±31·6 | | ±42·8 | ±22·1 | |
| | | ($n$=10) | | | (n=10) | | |
| | 3 | 139·1 | 281·7 | 49·4 | 138·7 | 338·8 | 40·9 |
| | | ±28·7 | ±28·3 | | ±39·9 | ±35·3 | |
| | | ($n$=10) | | | (n=8) | | |
| Large leg tumours (350–550 mm³) | 1 | 98·9 | 739·4 | 13·4 | 47·6 | 815·0 | 5·8 |
| | | ±43·3 | ±65·2 | | ±15·0 | ±74·5 | |
| | | ($n$=10) | | | (n=11) | | |
| | 3 | 84·3 | 456·6 | 18·5 | 28·8 | 437·5 | 6·5 |
| | | ±21·3 | ±58·6 | | ±13·8 | ±43·4 | |
| | | ($n$=10) | | | (n=9) | | |
| Very large leg tumours (700–1100 mm³) | 1 | 118·4 | 924·6 | 12·8 | | nd | |
| | | ±37·8 | ±77·8 | | | | |
| | | ($n$=9) | | | | | |
| | 3 | 125·5 | 508·8 | 24·7 | | nd | |
| | | ±33·2 | ±45·7 | | | | |
| | | ($n$=8) | | | | | |
| Small flank tumours (60–140 mm³) | 1 | 387·0 | 665·7 | 58·1 | | nd | |
| | | ±57·8 | ±73·3 | | | | |
| | | ($n$=10) | | | | | |
| | 3 | 219·9 | 432·1 | 50·9 | | nd | |
| | | ±46·3 | ±50·5 | | | | |
| | | ($n$=10) | | | | | |
| Large flank tumours (350–550 mm³) | 1 | 265·1 | 874·1 | 30·3 | | nd | |
| | | ±69·1 | ±50·2 | | | | |
| | | ($n$=8) | | | | | |
| | 3 | 179·9 | 511·5 | 35·2 | | nd | |
| | | ±59·5 | ±110·5 | | | | |
| | | ($n$=8) | | | | | |

Figures are mean ±2 s.e. of $n$ determinations.
nd = not determined.

TABLE V.—*Results of analysis of variance on log tumour/plasma ratios for* 1*h and* 3*h data from Table IV*

| | 1h data | | 3h data | |
|---|---|---|---|---|
| Effect | F ratio | *P* | F ratio | *P* |
| Heat | 8·2 | <0·01 | 22·9 | <0·001 |
| Size | 138·9 | <0·001 | 93·0 | <0·001 |
| Heat × size | 1·51 | >0·2 | 10·2 | <0·01 |
| Site | 18·4 | <0·001 | 13·0 | <0·001 |
| Size | 81·0 | <0·001 | 64·6 | <0·001 |
| Site × size | 13·5 | <0·001 | 11·7 | <0·01 |

show that there is no significant difference ($P > 0{\cdot}1$) in MISO concentrations between the centres and peripheries of tumours having had the same treatment, *i.e.* either heated or controls. However, the difference reported above between heated and unheated tumours is again seen. There is a significant difference in tumour concentrations, for both peripheral and central portions, between heated and unheated tumours ($P < 0{\cdot}01$).

### *Effect of the presence of a temperature probe on tumour levels of MISO*

Having previously reported that the presence of a temperature probe can affect

TABLE VI.—*Comparison of MISO and Ro* 05-9963 *levels* ($\mu g/g$) *in central and peripheral areas of leg tumours, heated and unheated, for a* 1·0 *g/kg dose of MISO*

| | | Control ($n=10$) | | Heat ($n=9$) | |
|---|---|---|---|---|---|
| | | MISO | Ro 05-9963 | MISO | Ro 05-9963 |
| Periphery | mean | 729 | 24·6 | 354 | 16·9 |
| | 2 × s.e. | 203 | 5·8 | 91 | 2·7 |
| Centre | Mean | 694 | 16·3 | 317 | 14·7 |
| | 2 × s.e. | 160 | 4·7 | 120 | 4·1 |

the survival of tumours treated with MISO and heat (Honess *et al.*, 1978) we investigated the possibility that the bleeding caused by the probe could cause higher MISO levels in monitored than unmonitored tumours. Experiments were carried out on male mice bearing small leg tumours heated for 1 h after administration of 1·0 g/kg MISO. They had been anaesthetized with Sagatal 10 min before receiving MISO. Tumours were excised immediately after heating, and the results indicated no significant difference between monitored and unmonitored tumours ($P > 0·1$); hence the extra cell killing in the presence of the probe cannot be attributed to higher MISO levels.

### DISCUSSION

In this paper we have shown that sodium pentobarbitone anaesthesia does not alter the plasma kinetics of MISO in normal mice. We have also shown that the presence of a tumour does not affect the plasma MISO levels. Neither the anaesthetic nor the tumour had a significant effect on the half-life or AUC. Also the oxidative demethylation to the Ro 05-9963 metabolite was unchanged. We therefore proceeded to investigate the effect of local hyperthermia on plasma and tumour kinetics of MISO, being as certain as possible that no differences that might be observed could be attributed to an anaesthetic artefact, anaesthesia being unavoidable in our current method of tumour heating.

Local hyperthermia was found to have very little effect on the plasma pharmacokinetics of MISO. There was, however, a tendency for the plasma MISO $t_{\frac{1}{2}}$ in locally heated animals to be rather longer at the high dose (1·0 g/kg) of MISO, but this was not significant in 2/3 experiments, nor at the low dose (100 mg/kg). The increase in $t_{\frac{1}{2}}$ was due to higher plasma levels at the later times (8–12 h), when no more than 20% of the peak drug concentration remained. As a result, the MISO AUC was not affected. Previous reports have suggested that the toxicity of MISO is related to the AUC (Dische *et al.*, 1977; Workman, 1979). Our finding that hyperthermia does not affect the plasma AUC of MISO is interesting in view of the increased toxicity of the drug by this treatment (Overgaard, 1979; unpublished results). This suggests that other factors may also be involved in the toxicity. Our finding that locally applied hyperthermia, which also caused a systemic temperature rise of between 2 and 3°C, has very little effect on the pharmacokinetics of MISO is essentially similar to that of Mimnaugh (1978) on Adriamycin in rabbits. In that study the core temperature of hyperthermic animals was 2·5°C higher than that of controls.

In contrast to the finding that local heating had little effect on systemic MISO pharmacokinetics, this treatment was found to reduce the MISO tumour levels, differences of up to 70% being found in the early samples. Other studies on tissue uptake of drug at raised temperatures deal with normal tissues, *e.g.* dog leg (Rochlin *et al.*, 1961) and rabbit skeletal muscle and duodenum (Mimnaugh, 1978) and they both demonstrate a higher level in the heated normal tissue. It seems unlikely that the reduction in MISO concentration could be due to increased oxidative metabolism in the tumour (which is on average 2°C hotter than the maximum core temperature) because correspondingly higher Ro 05-9963 levels might be expected, yet these were found to be unchanged. However, we cannot rule out the possibility that more rapid metabolism by other routes may be responsible. It is also possible that the decreased MISO concentrations in heated tumours are lower due to a reduction in the blood supply. This might be caused by heat-induced oedema constricting the blood vessels in the leg; oedema is brought about within 30 min of immersion in a 44°C water bath. Alternatively, it may be caused by a reduction in blood flow to the heated tumour, associated with a drop in blood pressure caused by sodium pentobarbitone. We did show that in the absence of heat sodium pentobarbitone did not affect tumour MISO concentration, but were unable to compare heated tumours with and without the anaesthetic. Johnson *et al.* (1976) have measured blood pressure and tumour blood flow at different temperatures in CBA and WHT mice bearing leg tumours under sodium pentobarbitone anaesthesia. Observations were made on blood flow at 20 and 39°C. Whereas at 20°C the anaesthetic caused a slight decrease in blood flow followed by recovery, at 39°C it caused falls of between 5 and 20%. The authors speculate that the effect of sodium pentobarbitone on the blood flow at hyperthermic temperatures suggests that the lowered blood pressure decreases total tumour perfusion. If this is indeed the case in this work, then we have not contrived to avoid all anaesthetic artefacts.

Bleehen *et al.* (1977) have shown that cell survival, as measured by *in vitro* plating assay, is lower at the periphery of heated MISO-treated tumours than at the centre. We have shown that there is no difference between the MISO concentrations at the periphery and centre of such tumours. Thus the possibility that lower drug concentrations in hotter parts of the tumour can lower the overall concentration in heated tumours is eliminated. It appears that the difference is for the tumour as a whole, as would be indicated by the impedance of blood flow, by whatever mechanism.

The finding that the tumour drug concentration can be reduced by heating is important for drugs the *in vivo* cytotoxicity of which is enhanced by hyperthermia, such as MISO itself, cyclophosphamide (unpublished observations) and bleomycin (Marmor, 1979). In these cases, the potential effect must exceed the observed effect, since the heated tumours have received less drug than the controls. This must be considered in evaluating the exploitability of such an enhancement.

The observation that tumour size and implantation site in unheated tumours influence the MISO concentration in those tumours is also significant. It is perhaps to be expected that larger tumours would have lower total levels of drug, because part of their volume is composed of a necrotic centre with no intact blood supply, which will receive drug only by diffusion. Donelli *et al.* (1977) have shown in intramuscular Lewis lung tumours, for a variety of cytotoxic agents, that drug is mainly to be found in the viable part, with very little in the necrotic parts of the tumour. None the less it is surprising that the same increase in tumour size has more effect on the drug concentration in leg tumours than in flank tumours. This may be due to differences in geometry and hence the relative sizes of necrotic centres in leg and flank tumours with similar calculated volumes. The demonstration that, irrespective of site, large tumours have lower concentrations of drug than small ones may be of importance with regard to reports of investigations with other drugs where small tumours have shown greater sensitivity, estimated by growth delay, than large ones. This phenomenon has been reported for the response of the Lewis lung tumour to cyclophosphamide (Steel & Adams, 1975) and for the response of the P815X2 mastocytoma and the EMT6 tumour to

BCNU (Schenken, 1976; Twentyman, 1978). Perhaps the effect is partly a reflection of the actual tumour drug dose as well as the usually accepted explanation in terms of proliferative state.

The demonstration that MISO levels are the same in monitored and unmonitored tumours shows that the greater cell killing in heated monitored tumours treated with MISO (Honess *et al.*, 1978) cannot be attributed to higher drug levels due to intra-tumoural haemorrhage. It may therefore be caused by better temperature equilibration of the monitored tumours due to conduction along the metal probe within the tumour, or by direct mechanical damage.

The main conclusions from this work are that the pharmacokinetics of MISO are unaffected by sodium pentobarbitone anaesthesia and very slightly affected by hyperthermia at a 44°C water bath temperature for 1 h. However, tumour levels of MISO are reduced. Also tumour levels of MISO in unheated tumours vary according to size and site. It seems unlikely that this phenomenon should be confined to MISO and, if it were shown to occur for other drugs also, it would at least partially account for some of the reports of variation in tumour chemosensitivity with size.

We thank Ms Jane Donaldson for expert technical assistance, Mr L. S. Freedman for statistical advice and Dr C. Smithen of Roche Products Limited for supplies of nitroimidazoles.

### REFERENCES

BALLARD, B. E. (1974) Pharmacokinetics and Temperature. *J. Pharma. Sci.*, **63**, 1345.

BLEEHEN, N. M., HONESS, D. J. & MORGAN, J. E. (1976) The interaction of the hypoxic cell sensitiser Ro 07-0582 and hyperthermia *in vitro* and *in vivo*. In *Int. Sympl on Radiobiol. Res., needed for improvement of radiotherapy*. Vienna: International Atomic Energy Agency. p. 211.

BLEEHEN, N. M., HONESS, D. J. & MORGAN, J. E. (1977) Interaction of hyperthermia and the hypoxic cell sensitiser Ro 07-0582 on the EMT6 mouse tumour. *Br. J. Cancer*, **35**, 299.

BROWN, J. M. (1975) Selective radiosensitisation of the hypoxic cells of mouse tumours with the nitroimidazoles metronidazole and Ro 07-0582. *Radiat. Res.*, **64**, 633.

DONELLI, M. G., BROGGINI, M., COLOMBO, T. & GARATTINI, S. (1977) Importance of the presence of necrosis in studying drug distribution within tumour tissue. *Eur. J. Drug Metab. Pharmacokinet.*, **2**, 63.

DISCHE, S., SAUNDERS, M. I., LEE, M. E., ADAMS, G. E. & FLOCKHART, I. R. (1977) Clinical testing of the radiosensitiser Ro 07–0582: Experience with multiple doses. *Br. J. Cancer*, **35**, 567.

DOUGLAS, B. G. & FOWLER, J. F. (1976) The effect of multiple small doses of X-rays on skin reactions in the mouse and a basic interaction. *Radiat. Res.*, **66**, 401.

GEORGE, K. G., HIRST, D. G. & MCNALLY, N. J. (1977) Effect of hyperthermia on cytotoxicity of the radiosensitizer Ro 07-0582 in a solid mouse tumour. *Br. J. Cancer*, **35**, 372.

HENDRY, J. M. (1978) Radionecrosis of normal tissues. Studies on mouse tails. *Int. J. Radiat. Biol*, **33**, 47.

HONESS, D. J., MORGAN, J. E. & BLEEHEN, N. M. (1978) The hyperthermic potentiation of the cytotoxic effect of misonidazole on the EMT6 mouse tumour: Relevance of *in vitro* measurement of *in vivo* effect. *Br. J. Cancer*, **37**, Suppl. III, 173.

HORNSEY, S., MYERS, R. & ANDREOZZI, U. (1977) Differences in the effects of anaesthesia on hypoxia in normal tissues. *Int. J. Radiat. Biol.*, **32**, 609.

JOHNSON, R., FOWLER, J. F. & ZANELLI, G. D. (1976) Changes in mouse blood pressure, tumour blood flow and tumour temperatures following nembutal or urethane anaesthesia. *Radiology*, **118**, 697.

KEIZER, H. J. & VAN PUTTEN, L. M. (1976) The radioprotective action on bone marrow CFU during immobilisation of mice. *Radiat. Res.*, **66**, 326.

MARMOR, J. B. (1979) Interactions of hyperthermia and chemotherapy in animals. *Cancer Res.*, **39**, 2269.

MIMNAUGH, E. G., WARING, R. W., SIKIC, B. I. & 5 others (1978). Effect of whole-body hyperthermia on the disposition and metabolism of adriamycin in rabbits. *Cancer Res.*, **38**, 1420.

MOHINDRA, J. K. & RAUTH, A. M. (1976) Increased cell killing by metronidazole and nitrofurazone of hypoxic compared to aerobic mammalian cells. *Cancer Res.*, **36**, 930.

OVERGAARD, J. (1979) Effect of local hyperthermia on the acute toxicity of mizonidazole in mice. *Br. J. Cancer*, **39**, 96.

PATERSON, E. & MATTHEWS, J. J. (1951) Protective effect of ethyl alcohol on irradiated mice. *Nature*, **168**, 1126.

PEACOCK, J. H. & STEPHENS, T. C. (1978) Influence of anaesthetics on tumour cell kill and repopulation in B16 melanoma treated with melphalan. *Br. J. Cancer*, **35**, 725.

ROCHLIN, D. B., THAXTER, T. H., DICKERSON, A. G. & SHINER, J. (1961) The effect of tissue temperature on the binding of alkylating agents in the isolation perfusion treatment of cancer. *Surg. Gynec. Obstet.*, **113**, 555.

SCHENKEN, L. L. (1976) Proliferative character and growth modes of neoplastic disease as determinants of chemotherapeutic efficiency. *Cancer Treat. Rep.*, **60**, 1761.

SHELDON, P. W., HILL, S. A. & MOULDER, J. E. (1977) Radioprotection by pentobarbitone sodium of a murine tumour *in vitro*. *Int. J. Radiat. Biol.*, **32**, 571.

SLADEK, N. E., DOMEGER, B. E., MERRIMAN, R. L. & BIOPHY, G. T. (1978) Differential effects of Walker 256 carcinosarcoma cells growing subcutaneously, intramuscularly or intraperitoneally on hepatic microsomal mixed-function oxygenase activity. *Drug Metab. Dispos.*, **6**, 412.

STEEL, G. G. & ADAMS, K. (1975) Stem cell survival and tumour control in the Lewis lung carcinoma. *Cancer Res.*, **35**, 1530.

STRATFORD, I. J. & ADAMS, G. E. (1977) Effect of hyperthermia on differential cytotoxicity of a hypoxic cell radiosensitiser, Ro 07-0582 on mammalian cells *in vitro*. *Br. J. Cancer*, **35**, 307.

SUTHERLAND, R. M., KOCH, C. J., BIAGLOW, J. E. & SRIDHAR, R. (1976) Potential chemotherapeutic drugs with selective toxicity for hypoxic cells. *Proc. Am. Ass. Cancer Res.*, **67**, 883.

TWENTYMAN, P. R. (1978) Sensitivity to 1,3-Bis(2-chloroethyl)-1-nitrosourea and 1-(2-chloroethyl)-3-(4-methylcyclohexyl)-1-nitrosourea of the EMT6 tumour *in vivo* as determined by both tumour volume response and *in vitro* plating assay. *Cancer Res.*, **38**, 2395.

TWENTYMAN, P. R. & BLEEHEN, N. M. (1975) Studies of "potentially lethal damage" in EMT6 mouse tumour cells treated with bleomycin either *in vitro* or *in vivo*. *Br. J. Cancer*, **32**, 491.

WATSON, J. V. (1976) The cell proliferation kinetics of the EMT6/M/AC mouse tumour at four volumes during unperturbed growth. *Cell Tissue Kinet.*, **9**, 147.

WORKMAN, P. (1979) Effects of pretreatment with phenobarbitone and phenytoin on the pharmacokinetics and toxicity of misonidazole in mice. *Br. J. Cancer*, **40**, 335.

WORKMAN, P., LITTLE, C. J., MARTEN, T. R. & 4 others (1978) Estimation of the hypoxic cell sensitiser misonidazole and its O-demethylated metabolite in biological materials by reversed-phase high-performance liquid chromatography. *J. Chromatogr.*, **147**, 507.